# Case Report: Preimplantation Genetic Testing and Pregnancy Outcomes in Women With Alport Syndrome

**DOI:** 10.3389/fgene.2021.633003

**Published:** 2021-02-09

**Authors:** Wei-Hui Shi, Mu-Jin Ye, Song-Chang Chen, Jun-Yu Zhang, Yi-Yao Chen, Zhi-Yang Zhou, Ning-Xin Qin, Xuan-You Zhou, Nai-Xin Xu, Zi-Ru Jiang, Jing Lin, He-Feng Huang, Chen-Ming Xu

**Affiliations:** ^1^International Peace Maternity and Child Health Hospital, School of Medicine, Shanghai Jiao Tong University, Shanghai, China; ^2^Shanghai Key Laboratory of Embryo Original Diseases, Shanghai, China

**Keywords:** X-linked Alport syndrome, preimplantation genetic testing, haplotype analysis, prenatal diagnosis, pregnancy, proteinuria

## Abstract

**Background:**

Alport syndrome, a monogenic kidney disease, is characterized by progressive hemorrhagic nephritis, sensorineural hearing loss, and ocular abnormalities. Mutations in *COL4A5* at Xq22 accounts for 80–85% of X-linked Alport syndrome patients. Three couples were referred to our reproductive genetics clinic for prenatal or preconception counseling.

**Methods:**

Prenatal diagnoses were performed by amplifying targeted regions of *COL4A5*. Targeted next-generation sequencing (NGS)-based haplotype analysis or karyomapping was performed in two patients. Pregnancy outcomes in the three patients were collected and analyzed. Published Alport syndrome cases were searched in Pubmed and Embase.

**Results:**

Prenatal diagnoses in two cases showed one fetus harbored the same pathogenic mutation as the proband and the other was healthy. The couple with an affected fetus and the patient with a family history of Alport syndrome chose to take the preimplantation genetic testing (PGT) procedure. One unaffected embryo was transferred to the uterus, and a singleton pregnancy was achieved, respectively. Two patients presented non-nephrotic range proteinuria (<3 g/24 h) during pregnancy and the three cases all delivered at full-term. However, published Alport cases with chronic kidney disease or proteinuria during pregnancy were came with a high rate (75%) of adverse maternal and fetal outcomes.

**Conclusion:**

The PGT procedure performed in this study was proven to be practicable and might be expanded to be applied in other monogenic diseases. Moderate or severe renal impairments in Alport syndrome were strongly associated with adverse maternal and fetal outcomes, and baseline proteinuria was a potential predictor for pregnancy outcomes of Alport syndrome as other kidney diseases.

## Introduction

Alport syndrome is an inherited monogenic kidney disease, first reported in a single big pedigree over 25 years (1902–1927), associated with progressive hemorrhagic nephritis and extraordinary malformations including sensorineural hearing loss and ocular abnormalities, such as anterior lenticonus, cataract, and maculopathy (Guthrie, 1902; Alport, 1927; Cohen et al., 1961; Colville and Savige, 1997). The typical triad of Alport syndrome symptoms is caused by structural damage of collagen type IV, an important component of basement membranes, especially in glomeruli (Kashtan et al., 2018). Three genes, *COL4A3*, *COL4A4*, and *COL4A5*, encode the α-chains (α3, α4, and α5, respectively) of collagen type IV, mutations in any of which may prevent the α3-α4-α5 network forming and the kidney glomerular basement membrane (GBM) will be functionally impaired (Kalluri et al., 1997). The prevalence of Alport syndrome is estimated at 1:50,000 of the population, approximately 80–85% of which are inherited in an X-linked dominant trait, associated with mutations in *COL4A5*; about 15% are autosomal recessive and rare (<5%) are autosomal dominant Alport syndrome, both resulting from *COL4A3* or *COL4A4* mutations (Keithi-Reddy and Kalluri, 2008).

*COL4A5* (NM_000495.4), consisting of 51 exons, is located at Xq22 and associated with X-linked Alport syndrome. Until now, more than one thousand *COL4A5* mutations covering variable types have been identified, however, without any mutational hot spots. One of the most conspicuous features of X-linked Alport syndrome is the impairment of renal function, including hematuria, albuminuria, and proteinuria, which usually progresses to end-stage renal disease (ESRD) in almost all male patients and in 10–30% females (Savige et al., 2016). Similar tendencies are also showed in the other two diagnostic criteria of X-linked Alport syndrome where male patients always show a higher risk of ocular changes and hearing loss than females (Hertz et al., 2015). In addition to gender differences in these phenotypes, male patients with X-linked Alport syndrome apparently display solid genotype-phenotype correlations. Mutations at 5′ end region of *COL4A5* always induce earlier age of ESRD onset and higher occurrence of ocular changes and hearing loss in males, but it is not obvious in females (Yamamura et al., 2017). Hence, females with Alport syndrome always are not recognized until they give birth to a male child or a patient is diagnosed in their pedigrees.

Currently, an effective treatment for Alport syndrome, angiotensin-converting enzyme (ACE) inhibitors, is recommended initiating once proteinuria, or even before, to slow down the progression of ESRD and prolong the life expectancy (Savige, 2014). Therefore, prenatal diagnosis is essential for families with Alport syndrome affected members, upon which their affected offsprings could receive treatments in time. Nevertheless, medical therapies are not specific cures or long-term protectors. ESRD is still an inevitable outcome of X-linked Alport syndrome (Gross et al., 2012). As an alternative to prenatal diagnosis, preimplantation genetic testing (PGT) is a procedure that genetic information of the embryos is analyzed with one or more cells from cleavage-stage embryos or blastocytes prior to transferring, involving multiple assisted reproductive techniques, including *in vitro* fertilization (IVF), intracytoplasmic sperm injection (ICSI), embryo cryopreservation and single-cell genetic testing (Sermon et al., 2004; Dahdouh et al., 2015). PGT aims to prevent the transmission of genetic defects, particularly in couples who reject abortion for the sake of religions or moralities (Braude et al., 2002). Since the first successful application of PGT in X-linked disorders was reported in 1990, it has been sophisticatedly used in diagnoses of monogenic diseases, sex-linked disorders, aneuploidy, and chromosomal rearrangements (Handyside et al., 1990; Spits and Sermon, 2009). And since the first Alport syndrome referral in 1995, PGT for monogenic kidney diseases has steadily increased (Snoek et al., 2020). However, it is ethically controversial in the application of PGT for late-onset diseases (Huntington disease or Alzheimer disease), cancer predisposition genes (*BRCA1*, *BRCA2*, and *p53* tumor suppressor gene) and HLA typing (leukemia, Fanconi anemia, and severe combined immunodeficiency syndrome) (El-Toukhy et al., 2008). The attitude to these ethical debates differ from country to country because of different regulation of PGT worldwide.

Pregnancy in women with Alport syndrome has been followed up in several cases (Matsubara et al., 2009; Mehta et al., 2013; Alessi et al., 2014; Brunini et al., 2018), but the maternal and fetal risks of Alport syndrome are still indistinct. In this study, we performed prenatal diagnosis or/and PGT in three Chinese families with Alport syndrome. Meanwhile, the impacts of Alport syndrome on maternal and fetal outcomes and pregnancy on the disease progression were evaluated in our three and other published cases.

## Materials and Methods

### Sanger Sequencing and Data Analysis

Genomic DNA of the four families, including probands in each pedigree, were extracted from peripheral blood samples according to standard procedures. Target regions of the *COL4A5* were amplified with specific primers (Supplementary Table 1) and were subsequently sequenced on an Applied Biosystems 3500Dx sequencer. The primers were designed with the Primer 3 Web (Untergasser et al., 2012). Frequencies of variants were checked in Exome Aggregation Consortium^1^. The interpretation and classification of variants were based on the ACMG guideline (Richards et al., 2015). The functional predictions of detected variants were achieved with the dbNSFP database (Liu X. et al., 2016). To validate the absence of contamination, blank controls were processed under identical conditions.

### Identity Testing and Haplotype Analysis

Short tandem repeat (STR) markers were detected for potential maternity contamination analysis and identity testing with an identification detection kit (R1004T; GENESKY, Shanghai, China) according to the instruction manuals. The whole genomes of lymphocytes of the families or single blastomeres of embryos were amplified with a Qiagen whole genome amplification kit (150345#REPLI-g Single Cell Kit (96), QIAGEN/A) and sequenced by karyomapping (Natesan et al., 2014) or by targeted NGS technique, an array-based gene chip described previously (Chen et al., 2016). Based on informative SNPs, which is the same location in the parents but homozygous in one and heterozygous in the other, co-segregating with the identified mutation, haplotype analysis was carried out to confirm the carrier status in the family and the inheritance of embryos. The variant was described adhering to the Human Genome Variation Society (HGVS) nomenclature (version 15.11) (den Dunnen et al., 2016).

### Preimplantation Genetic Testing (PGT) for Monogenic Diseases Procedure

Controlled ovarian stimulations were administrated following the gonadotropin-releasing hormone (GnRH) antagonist protocol, the multiple-dose flexible regimen (Al-Inany et al., 2016). Intracytoplasmic sperm injection (ICSI) was taken on metaphase II (MII) oocytes, injecting an immobilized sperm with micropipettes as the standard procedure (Devroey and Van Steirteghem, 2004). These single blastomere biopsies were applied with whole genome amplification, followed by karyomapping or targeted NGS technique for genetic testing mentioned above. Blank controls were set for checking the extraneous DNA contamination. Biopsied embryos were cryopreserved with vitrification methods for later embryo transfer cycles. Diagnosis of clinical pregnancy was considered to be the presence of a gestational sac by ultrasound after 30–32 days after embryo transfer. Routine prenatal care of pregnant women after PGT was taken on in our hospital. Fully informed consents were signed by our patients, and the study was approved by the Ethics Committee of the IPMCH of Shanghai Jiao Tong University School of Medicine before all the following procedure and adhered to the Declaration of Helsinki.

### Prenatal Diagnosis

Ultrasonography was performed as routine throughout the gestations of our consultants. For genetic disorders, the invasive prenatal genetic diagnosis was progressed on fetal sampling obtained from chorionic villus sampling or amniocentesis. Genomic DNA of fetal sampling was extracted and sequenced as mentioned above. Blood pressure, serum creatinine, and urinalyses were tested to monitor the maternal kidney function during gestations.

### Search Strategy for Reported Pregnancies in Alport Syndrome Cases

We performed a literature search for pregnancies in Alport syndrome cases in PubMed^2^ and Embase^3^, with keywords “Alport syndrome,” “COL4A3 or COL4A4 or COL4A5,” and “women or woman or female or pregnant.^∗^” We only included articles in English and Chinese.

## Case Presentation

**FIGURE 1:**
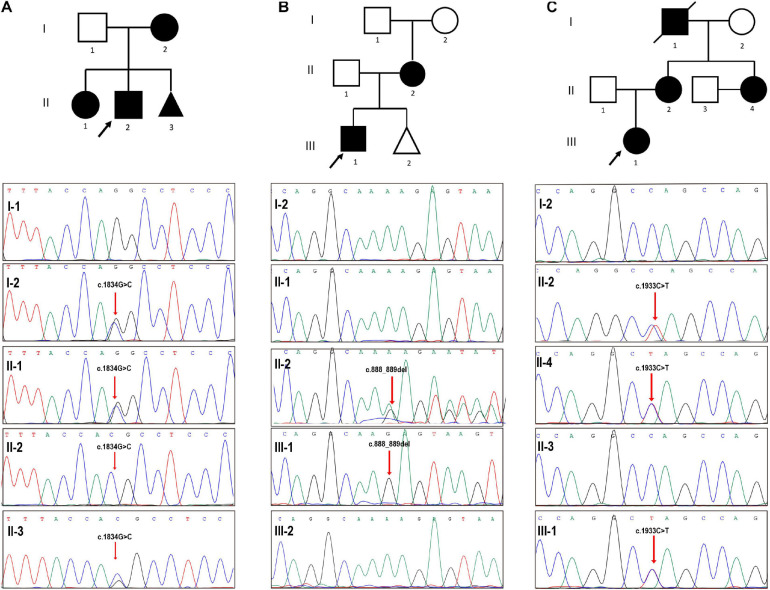
Validation of three pedigrees with *COL4A5* variants. **(A)** The proband (II-2), a 3-year-old boy, had been confirmed as an X-linked Alport syndrome patient with a pathogenic *COL4A5* mutation (NM_000495.4 (*COL4A5*): c.1834G > C) and manifested with hematuria. Prenatal diagnosis suggested that the fetus (II-3) harbored the same *COL4A5* mutation. **(B)** Sanger sequencing validated a hemizygous mutation in *COL4A5* (NM_000495.4 (*COL4A5*): c.888_889del) in the proband (III-1), manifesting as microscopic hematuria and albuminuria without edema, and his mother, while the fetus was not inherited the mutation. **(C)** The red arrow points to the mutated locus showing that our patient (II-4) harbored the same pathogenic mutation (NM_000495.4 (*COL4A5*): c.1933C > T) with the proband suffering from hematuria, her 3-year-old nephew. Black arrows indicate the probands in the three pedigrees.

Three couples were referred to the reproductive genetics clinic of the International Peace Maternity and Child Health Hospital (IPMCH) for preconception or prenatal counseling. Two naturally pregnant patients (Table 1, Nos. 1 and 2), 37 and 41 years old respectively, had given birth to children affected with Alport syndrome, and one 32-year-old patient (Table 1, Nos. 3) had a history of ectopic pregnancy and family history of Alport syndrome. Pathogenic COL4A5 mutations (c.1834G > C; c.888_889del; c.1933C > T) were validated in the three women by Sanger sequencing (Figure 1). Prenatal genetic testings were performed on two pregnant patients (patient Nos. 1 and 2) through chorionic villus or amniocentesis samplings on the basis of the genetic testing result. Sanger sequencing, without contamination detected with short tandem repeat (STR) markers (Supplementary Table 2), showed the fetus of patient No. 1 harbored the same pathogenic mutation as the proband, while the fetus of patient No. 2 was healthy (Figure 1). Although heterozygous COL4A5 mutations always lead to a late-onset renal insufficiency and milder phenotype than hemizygous mutations, the couple (patient No. 1) ultimately chose to terminate the pregnancy and requested PGT to have a healthy baby. For late-onset diseases, PGT might be challenging to be accepted in some countries because affected person were normal and healthy in their four or five decades or even in their full life, while supports believe that PGT is applicable for those who are unwilling to bear the burden imposed by the eventual fate in late-onset diseases (Sermon et al., 2004). The artificial abortion operation was conducted after informed consent was signed.

**TABLE 1:** Pathogenic variants detected in three pregnant patients with Alport syndrome.

Patient	Pathogenic gene	Transcript ID	DNA variant	Amino acid changes	Classification of variants^#^
No. 1	*COL4A5*	NM_000495.4	c.1834G > A	p.(Gly612Arg)	Likely pathogenic
No. 2	*COL4A5*	NM_000495.4	c.888_889del	p.(Gly298*)	Pathogenic
No. 3	*COL4A5*	NM_000495.4	c.1933C > T	p.(Gln645*)	Pathogenic

^#^The interpretation and classification of variants was based on ACMG guideline (Richards et al., 2015). *Termination of protein translation.

## Results

### PGT in Two Pedigrees

Two patients (Table 1, patient Nos. 1 and 3) eagerly opted to PGT for unaffected offsprings. Following the gonadotropin-releasing hormone (GnRH) antagonist protocol, oocytes of the two patients were retrieved and fertilized by ICSI. Embryo biopsy following by targeted NGS sequencing (patient No. 1) or karyomapping (patient No. 3) with informative SNPs, showed that five embryos in patient No. 1 and one embryos in patient No. 3, respectively, inherited the mutation haplotype from their mothers. Following Sanger sequencing of the *COL4A5* mutations corroborated the targeted NGS sequencing results (Supplementary Figure 1). The blank controls indicated no contamination in the procedure. Finally, one unaffected embryo was transferred to the patients’ uterus in each PGT cycle.

### Follow-Up of Pregnancies

**FIGURE 2:**
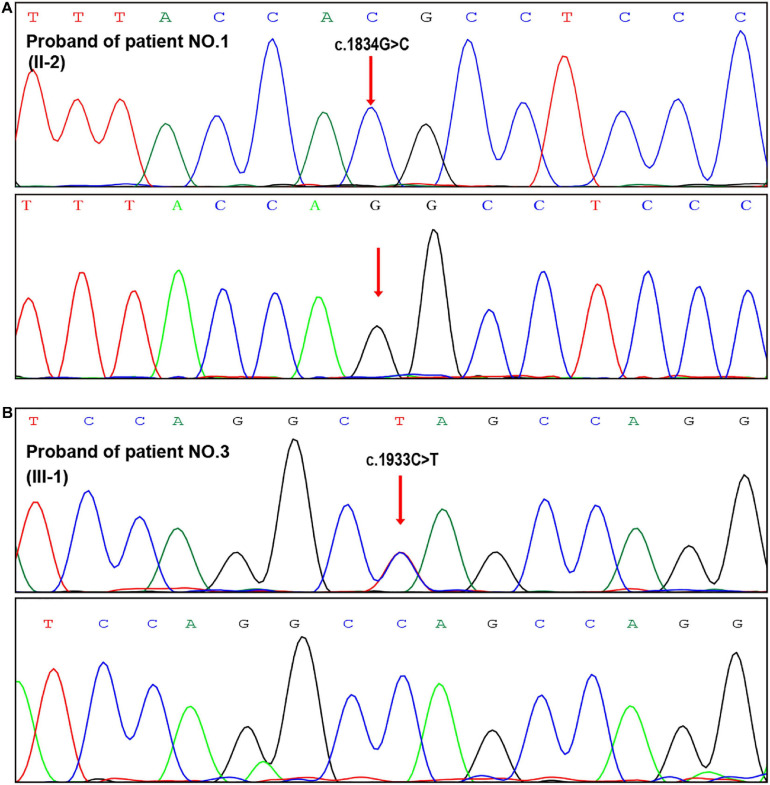
Prenatal genetic diagnosis after PGT. **(A,B)** Sanger sequencing of the fetal samplings from amniotic fluid indicated normal genotypes in the two PGT cases (patient Nos. 1 and 3).

We followed up the three patients throughout their gestations and 6 months after their deliveries finding that their maternal blood pressures were all in normal ranges (Table 2). Since patient No. 1 was diagnosed as an Alport syndrome patient by our genetic testing, microscopic hematuria has been constantly observed, which was the only renal manifestation before her artificial abortion operation. During pregnancy period, proteinuria was presented in the second trimester of the patient and gradually increased to 1.22 g/24 h in the third trimester, while the hematuria did not aggravate. Moreover, the patient suffered from premature membrane rupture at 39 weeks’ gestation but no occurrence of fever and infection before labor. Patient Nos. 2 and 3 both had microscopic hematuria prior to their pregnancies but with normal renal function. However, patient No. 3 presented with urinary protein at 30 weeks’ gestation, which increased to 1.57 g/24 h before delivery. In view of mild proteinuria and microscopic hematuria, the patients were all hospitalized transiently and observed intensively without any medications at conception. After prenatal diagnoses confirming the results of PGT (Figure 2), all babies were born at full terms and healthy.

**TABLE 2:** Maternal and fetal outcomes of the three Alport syndrome patients.

Patient	Age (years)	Before pregnancy				During pregnancy				After delivery				Gestational weeks	Delivery type	Birth weight(g)/ Gender
		Proteinuria (g/24 h)	Blood pressure	Renal function	Hematuria	Proteinuria (g/24 h)	Blood pressure (mmHg)	Renal function	Hematuria	Proteinuria (g/24 h)	Blood pressure	Renal function	Hematuria			
1	37	–	120/80	N	++	1.22	121/87	N	++	–	120/83	N	++	39+1	VD	3,685/F
2	41	–	119/76	N	++	–	120/70	N	++	–	122/75	N	++	39+5	CS	2,510/F
3	32	–	106/65	N	+++	1.57	104/67	N	+++	0.99	104/60	N	+++	39+3	VD	2,960/F

**N, Normal (serum creatinine level less than 0.7 mg/dL); VD, Vaginal delivery; CS, Cesarean section; −, Proteinuria is negative; +, Hematuria is positive.**

### Review of Reported Pregnancies in Alport Syndrome

Thirty pregnancies in different types of Alport syndrome had been reported in 20 cases (Matsuo et al., 2007; Zhang et al., 2007; Matsubara et al., 2009; Fabris et al., 2012; Matsubara and Muto, 2012; Saifan et al., 2012; Crovetto et al., 2013; Mehta et al., 2013; Alessi et al., 2014; Gerasimovska Kitanovska et al., 2016; Nishizawa et al., 2016; Yefet et al., 2016; Brunini et al., 2018; Drury et al., 2019; Supplementary Table 3). Most of the reported cases presented with normal blood pressure and renal function before conception. However, renal function in two patients (Patient 2/18 in Table 3) with pre-conceptional chronic kidney diseases tended to badly aggravate at conception accompanied with hypertension. They turned out with stillbirth and preterm labor at 25 and 29 weeks’ gestation, respectively. And they were treated with hemodialysis at postpartum. Another seven patients (35%, 7/20) (Patient 1/4/5/7/8/11/17) developed kidney impairment during pregnancy and six of them were complicated with high blood pressure. In eight pregnancies of these seven patients, preterm birth occurred in six gestations (75%, 6/8), the majority of which were resulted from preeclampsia or fetal growth delay leading to a high rate of cesarean sections (75%, 6/8). Worse outcomes were common in the overall thirty pregnancies, including preterm births (36.6%), preeclampsia (26.6%), fetal growth delay (10%), premature membrane rupture (3.3%) and cesarean sections (53.8%).

**TABLE 3:** Maternal outcomes of published Alport syndrome cases.

Patient Number	Before pregnancy				During pregnancy				After delivery			
	Proteinuria (g/24 h)	Blood pressure	Renal function	Hematuria	Proteinuria (g/24 h)	Blood pressure	Renal function	Hematuria	Proteinuria (g/24 h)	Blood pressure	Renal function	Hematuria
1	2	Normal	Normal^#^	+	7.14	Normal	Mild^$^	N/A	1.9	Normal	Mild^$^	N/A
2	2	High 230/130	CKD	–	15	High 242/109	ARF	–	Hemodialysis	N/A	RF	–
3^$^	2	Normal	Normal	+	2.22	Normal	Normal	N/A	1.5	Normal	Normal	N/A
3^$^	1	Normal	Normal	–	2	Normal	Normal	–	1.5	Normal	Normal	–
4^$^	N/A	N/A	N/A	N/A	14.1	high 162/111	Mild	N/A	N/A	High	Mild^$^	N/A
5	+	Normal	Normal	–	15	high 162/111	ARF	–	0.75	Normal	Mild^$^	N/A
6	N/A	N/A	N/A	N/A	7	Normal	Normal	–	0.99	Normal	Normal	–
7	N/A	N/A	N/A	N/A	3.26	High	Worsen	–	3.21	Normal	CKD	–
8*	0.8	Normal	Normal	–	20	High 140/80	Moderate	+	2	Normal	Mild^$^	–
8*	2	Normal	CKD	–	3.5	Normal	Mild^$^	–	2	Normal	Mild^$^	–
9	1.6	Normal	Normal	+	4.7 g/gCr	Normal	Normal	N/A	0.6 g/gCr	Normal	Normal	N/A
10*	–	Normal	Normal	+	–	Normal	Normal	+	–	Normal	Normal	+
10*	–	Normal	Normal	+	–	Normal	Normal	+	–	Normal	Normal	+
10*	–	Normal	Normal	+	–	Normal	Normal	+	–	Normal	Normal	+
10*	–	Normal	Normal	+	–	Normal	Normal	+	–	Normal	Normal	+
11*	+			+	–	–	–	–	–	–	–	–
11*	+			+	–	–	–	–	–	–	–	–
11*	+	N/A	N/A	+	+	High	Worsen	+	Peritoneal dialysis	N/A	CKD	+
12*	+	Normal	Normal	+	0.5–1.5	Normal	Normal	N/A	+	Normal	Normal	N/A
12*	+	Normal	Normal	N/A	3.6	Normal	Normal	N/A	0.63	Normal	Normal	N/A
12*	0.63	Normal	Normal	N/A	3.9	Normal	Normal	N/A	0.6	Normal	Normal	N/A
13	0.1	Normal	Normal	–	0.18	Normal	Normal	+	0	Normal	Normal	+
14	0.6	Normal	Normal	–	2.3	Normal	Normal	–	0.6	Normal	Normal	–
15*	1.9	Normal	Normal	–	9.3	Normal	Normal	–	2	Normal	Normal	–
15*	2	Normal	Normal	–	13	Normal	Normal	–	1.03	Normal	Normal	–
16	1.4	Normal	Normal	–	10.6	Normal	Normal	–	1.4	Normal	Normal	–
17	1.6	Normal	Normal	–	4.5	High 160/90	Mild^$^	–	0.9	Normal	Mild^$^	–
18	1.6	Normal	CKD	–	5	High 180/100	CKD		Hemodialysis	High 140/85	kidney transplant	–
19	n/a	N/A	N/A	N/A	6.94 g/gCr	Normal	Normal	+	2.3 g/gCr	Normal	Normal	–
20	–	Normal	Normal	+	+	Normal	Normal	+				

N/A, not available; CKD, Chronic kidney diseases; ARF, Acute renal failure; RF, Renal failure. ^#^Serum creatinine level less than 0.7 mg/dL. *Pregnancies of the same patient. ^$^Serum creatinine level over 0.7 mg/dL but less than 1.4 mg/dL. −, Proteinuria or hematuria is negative; +, Proteinuria or hematuria is positive.

For fetal outcomes of the seven patients developed kidney impairment during pregnancy, only four of the eight gestations were described in detail (Table 4). It was shown that all the five neonates (including a pair of twins) had low birth weights, two of whom died after birth. Furthermore, almost all reported patients presented proteinuria during pregnancy and worsen in the third trimester whenever proteinuria did exit or not before conception. Proteinuria in 65% patients progressively reached to the nephrotic range. In addition, hematuria was the other characteristic generally observed in pregnant Alport patients, whereas it was barely exacerbated during gestations.

**TABLE 4:** Fetal outcomes of published Alport syndrome cases.

Patient number	Age (years)	Preeclampsia	Fetal growth delay	Premature membrane rupture	Preterm birth	Gestational weeks	Delivery type	Birth weight (g)/Gender
1	26		√		√	34	CS	1700
2	29	√	√		Stillbirth	25	VD	400/F
3*	19					39	CS	2,862/M
3*	21					37	CS	2,458/F
4	20	√				N/A	CS	N/A
5	20	√			√	29	CS	N/A
6	38				√	34	CS	2,165/M
7	26	√			√	33	VD	2,400/M
8*	27	√			√	30	CS	880 (dead)
8*	29					39	CS	N/A
9	27					39	VD	3,216/F
10*	N/A					Fetal death of unknown cause		
10*	20					N/A	VD	N/A/F
10*	27					N/A	VD	N/A/F
10*	28					N/A	VD	N/A/F
11*	N/A					Spontaneous miscarriage		
11*	N/A					Spontaneous miscarriage		
11*	27	√			√	35	VD	N/A/M
12*	20					40	VD	3,500/M
12*	25					39	VD	3,100/M
12*	29				√	36	VD	2,686/F
13	33					38	VD	3,100/F
14	40			√		37	CS	3,210/F
15*	19				√	32	CS	1,830/F
15*	22				√	34	CS	2,630/F
16	16				√	36	CS	2,335/F
17	49	√	√		√	33	CS	1,720/M
								1,480/F(dead)
18	32	√			√	29	CS	1,200/F
								1,300/M
19	31					38	VD	3,201/F
20	29					37	Wait for delivery	

N/A, not available; VD, Vaginal delivery; CS, Cesarean section; F, Female; M, Male. *Pregnancies of the same patient.

## Discussion

For mothers of an affected Alport syndrome child as the maternity in this study, it is encouraged to refer to a clinical geneticist for carrier status ascertainment and a nephrologist for clinical assessments for the sake of the high possibility of harboring the same pathogenic mutation. Notably, our patients presented non-nephrotic proteinuria, especially in the last trimester of gestations. Regarding their stable conditions without hypertension and infection, they were not treated with any medications but observed intensively. Fortunately, the proteinuria turned out to diminish and vanish after the delivery.

Studies demonstrated that chronic kidney disease, resulted from different types of kidney diseases, was a significant risk for adverse pregnancy outcomes, including gestational hypertension, preeclampsia, eclampsia, maternal mortality, fetal growth restriction, preterm births, stillbirths, and low birth weight (Nevis et al., 2011; Zhang et al., 2015). Even mild renal impairment remained associated with adverse maternal and fetal outcomes in women without baseline hypertension and proteinuria (Piccoli et al., 2015). In published pregnant Alport syndrome cases, two patients with pre-conceptional chronic kidney diseases had a severely progressive worsening in renal function during pregnancy and received hemodialysis after delivery (Matsuo et al., 2007; Brunini et al., 2018). Three preterm born neonates of the two patients were all weighing less than 1,500 g, and two of the babies died in uterine or a few hours after birth. And seven Alport patients suffering from renal impairment at gestations also presented a high rate of preterm birth, preeclampsia, cesarean sections, fetal growth delay, and fetal low birth weight, similar to pregnancy outcomes in other types of kidney diseases (Ekbom et al., 2001; Liu Y. et al., 2016).

Proteinuria was commonly presented at conceptions in published cases with Alport syndrome, as well as in our patients. A large cohort study confirmed that baseline proteinuria (>1 g/24 h) in patients with normal renal function was a potential risk factor for adverse pregnancy outcomes, while minimal proteinuria (<1 g/24 h) in patients would not be a risk factor of pregnancy to renal function (Williams and Davison, 2008; Piccoli et al., 2015). Moreover, it always deteriorated in the second and third trimester. In published Alport syndrome patients, eight pregnancies with isolated proteinuria had a high rate of preterm birth (62.5%, 5/8). Hence, proteinuria in pregnancies should be attached attention and controlled well whenever it existed with or without renal dysfunction. Besides, hematuria could be generally observed in pregnant Alport patients but merely worsen during pregnancy, which was likely to not influence pregnancy outcomes.

It has been recommended in expert guidelines for the management of Alport syndrome that a genetic consultation for affected individuals in regard to the inheritance and available reproductive options, including prenatal and preimplantation genetic diagnosis, be considered preferably prior to any pregnancy and be conducted in a non-directive way (Savige et al., 2013). A survey of X-linked Alport syndrome families in China showed that approximately 80% of respondents would opt to terminate a pregnancy with a positive prenatal genetic diagnosis (Zhang et al., 2012). Despite of the fact that the safety of amniocentesis or chorionic villus sampling has been already confirmed with low fetal loss rates and premature deliveries (Shulman and Elias, 2013), it is hard to deny that the procedure may be a potential hazard for adverse pregnancy outcomes in terms of few researches reporting invasive prenatal diagnoses in pregnant women associated with Alport syndrome. Therefore fully informed consent before prenatal diagnoses and closely care during or after the performance is necessary for pregnant patients associated with Alport syndrome.

As an early form of prenatal diagnosis, PGT avoiding the application of therapeutic termination, which involves multiple complications ranging from excessive bleeding, cervical trauma and infections to future infertility, might be an alternative choice for those Alport syndrome affected families. In our study, the renal function of patient No. 1 were normal both in her trimesters of the natural gestation and the PGT cycle, which suggested that the PGT procedure might be not a risk factor of maternal outcomes in women with Alport syndrome. However, bigeminal pregnancies in Alport syndrome, reported by Brunini et al. (2018) were resulted from assisted reproductive technology, having distinctly bad fetal outcomes, including preterm birth, low birth weight and neonatal death. Consequently, transferred embryos should be restricted to one at each time for Alport syndrome patients, as the burden of maternal kidney function and the procedure of prenatal diagnosis after PGT were taken into consideration.

In conclusion, three pedigrees suffering from Alport syndrome were validated harboring pathogenic *COL4A5* mutations. Meanwhile, prenatal diagnosis or targeted NGS-based PGT was performed to prevent the transmission of the pathogenic mutations of *COL4A5* in their families. Further, our literature review about pregnancies in Alport syndrome showed that moderate or severe renal impairment was strongly associated with adverse maternal and fetal outcomes and proteinuria before conception or during pregnancy was a potential predictor for pregnancy outcomes as other kidney diseases. The PGT procedure, including GnRH antagonist protocol with low risk of ovarian hyperstimulation syndrome, targeted haplotype analysis based on targeted NGS or karyomapping and singleton frozen-thawed embryo transfer, was proven to be practicable and effective and could be expanded to other monogenic diseases.

## Data Availability Statement

The raw data supporting the conclusions of this article will be made available by the authors, without undue reservation.

## Ethics Statement

The studies involving human participants were reviewed and approved by the Ethics Committee of the IPMCH of Shanghai Jiao Tong University School of Medicine. Written informed consent to participate in this study was provided by the participants’ legal guardian/next of kin. Written informed consent was obtained from the individual(s), and minor(s)’ legal guardian/next of kin, for the publication of any potentially identifiable images or data included in this article.

## Author Contributions

W-HS, M-JY, Y-YC, Z-YZ, N-XQ, X-YZ, N-XX, Z-RJ, and JL carried out the experiments. J-YZ analyzed the data. S-CC made the figures. W-HS drafted the manuscript. C-MX and H-FH revised the manuscript. All authors approved the final version of the manuscript.

## Conflict of Interest

The authors declare that the research was conducted in the absence of any commercial or financial relationships that could be construed as a potential conflict of interest.

## Footnotes

1 http://exac.broadinstitute.org/

2 https://pubmed.ncbi.nlm.nih.gov/

3 https://www.embase.com/
