# Supplementary material for: Case Report: Preimplantation Genetic Testing and Pregnancy Outcomes in Women With Alport Syndrome
Source: Front Genet. 2021 Feb 9;12:633003. doi: 10.3389/fgene.2021.633003 (PMC7900551; doi:10.3389/fgene.2021.633003)
Supplement: Supplementary file 1 [file Data_Sheet_1.PDF]

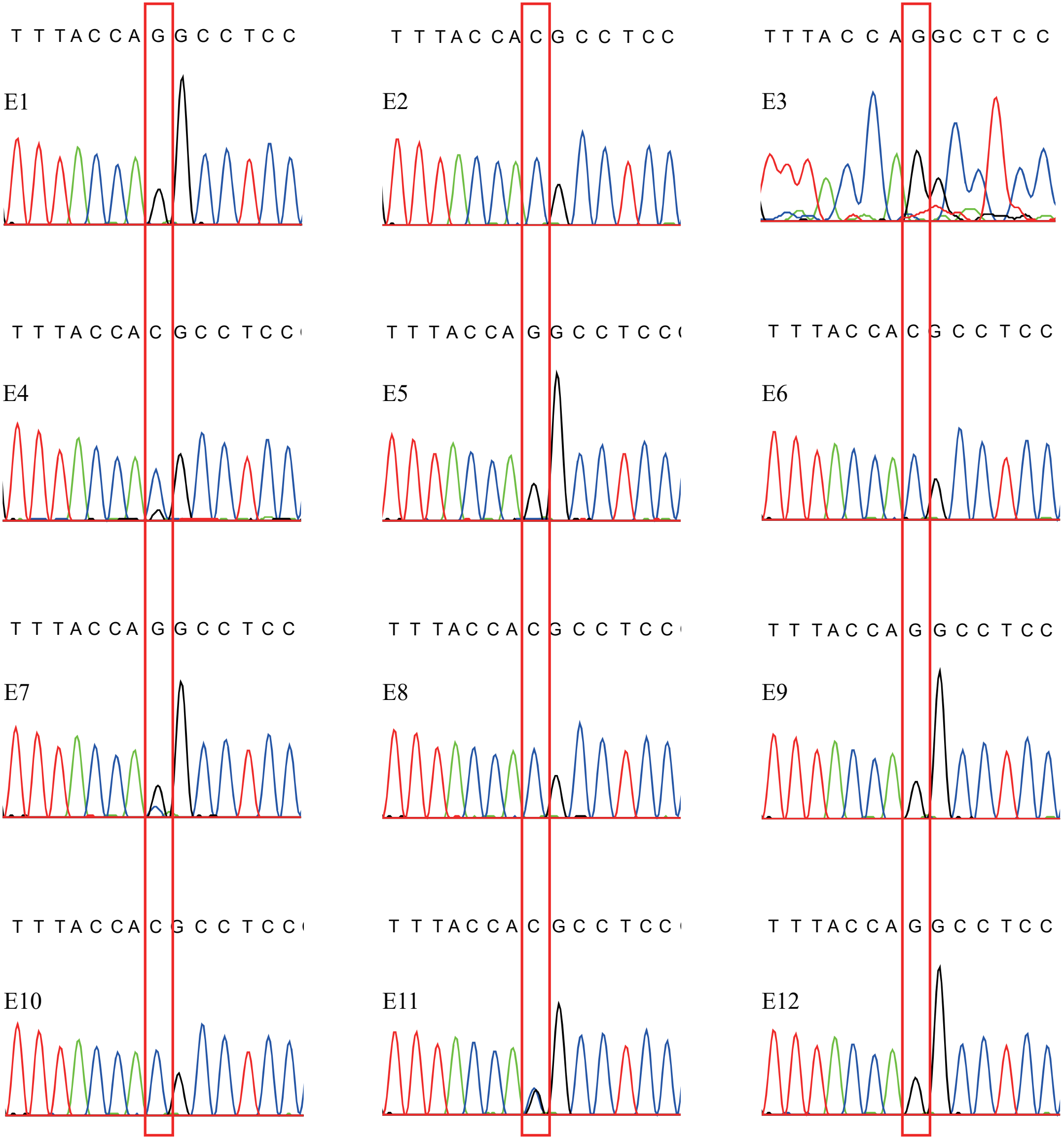

Supplemental figure 1. Sanger sequencing verified the *COL4A5* mutation in the twelve embryos (E1-E12) of patient NO.1.
