# Supplementary material for: Case Report: Preimplantation Genetic Testing and Pregnancy Outcomes in Women With Alport Syndrome
Source: Front Genet. 2021 Feb 9;12:633003. doi: 10.3389/fgene.2021.633003 (PMC7900551; doi:10.3389/fgene.2021.633003)
Supplement: Supplementary file 2 [file Data_Sheet_2.docx]

**Supplemental Table 1. Primers of *COL4A5* for Sanger sequencing**

| Patient | Primers |
| --- | --- |
| NO.1 | COL4A5-F1: 5’-TGGCTATATCCTTTCCCCAGT-3’  COL4A5-R1: 5’-GCCACACCTTGTATGCCTTT-3’ |
| NO.2 | COL4A5-F2: 5’-GGTACACAGTATCCCATGAACC-3’  COL4A5-R2: 5’-CATACTGCTTCTCTGCCACC-3’ |
| NO.3 | COL4A5-F3: 5’- AGGGAATATAGGGCCTATGGG-3’  COL4A5-R3: 5’- AGGAAGCCATGAGTAGCCAA-3’ |

**Supplemental Table 2. Short tandem repeat typing results of the family members**

| Marker | Patient NO.1 | | | | | | Patient NO.2 | | | | | | Patient NO.3 | | | | | |
| --- | --- | --- | --- | --- | --- | --- | --- | --- | --- | --- | --- | --- | --- | --- | --- | --- | --- | --- |
|  | Mother | | Father | | Fetus | | Mother | | Father | | Fetus | | Mother | | Father | | Fetus | |
|  | Allele 1 | Allele 1 | Allele 1 | Allele 2 | Allele 2 | Allele 1 | Allele 2 | Allele 1 | Allele 1 | Allele 2 | Allele 2 | Allele 1 | Allele 1 | Allele 2 | Allele 2 | Allele 1 | Allele 2 | Allele 1 |
| Amelo | X | X | X | Y | X | / | X | X | X | Y | X | / | X | X | X | Y | X | / |
| D13S317 | 9 | 14 | 9 | 12 | 9 | 9 | 8 | 10 | 9 | 9 | 8 | 9 | 8 | 10 | 10 | 11 | 10 | 11 |
| D7S820 | 9 | 10 | 11 | 12 | 9 | 11 | 10 | 10 | 11 | 12 | 10 | 12 | 11 | 12 | 12 | 12 | 12 | 12 |
| G4S0001 | 13 | 17.2 | 14 | 16 | 17.2 | 14 | 11 | 15 | 14 | 16.2 | 15 | 16.2 | 13 | 14 | 14 | 14 | 13 | 14 |
| G2S0002 | 24 | 25 | 24 | 25 | ? | 25 | 18.2 | 24 | 19.2 | 19.2 | 18.2 | 19.2 | 19.2 | 20.2 | 18.2 | 18.2 | 19.2 | 18.2 |
| D18S51 | ? | 19 | ? | 16 | ? | 19 | 17 | 22 | 13 | 16 | 22 | 16 | 16 | 16 | 20 | 22 | 16 | 20 |
| D8S1179 | 11 | 15 | 10 | 12 | ? | 10 | 10 | 10 | 11 | 14 | 10 | 11 | 12 | 15 | 13 | 14 | 12 | 13 |
| D2S1338 | 23 | 24 | 16 | 23 | 16 | 24 | 18 | 20 | 22 | 22 | 18 | 22 | 20 | 24 | 17 | 23 | 20 | 17 |
| G15S0001 | 13 | 16 | 11 | 11 | 11 | 13 | 13 | 14 | 11 | 16 | 11 | 14 | 9 | 12 | 11 | 11 | 12 | 11 |
| D16S539 | 10 | 12 | 11 | 12 | 10 | 12 | 9 | 12 | 8 | 11 | 9 | 11 | 9 | 13 | 10 | 13 | 13 | 13 |
| VWA | 17 | 19 | 14 | 17 | 19 | 17 | 14 | 14 | 16 | 17 | 14 | 17 | 17 | 17 | 17 | 19 | 17 | 19 |
| G7S0005 | 7 | 10 | 10 | 10 | 10 | 10 | 7 | 9 | 9 | 10 | 7 | 10 | 10 | 10 | 9 | 10 | 10 | 9 |
| G10S0001 | 17 | 19 | 15 | 19 | 15 | 19 | 19 | 20 | 19 | 19 | 19 | 20 | 19 | 19 | 19 | 19 | 19 | 19 |
| THO1 | 7 | 9 | 7 | 10 | 7 | 7 | 6 | 9 | 6 | 6 | 6 | 6 | 8 | 8 | 6 | 8 | 8 | 8 |
| D8S588 | 8 | 10 | 10 | 13 | 10 | 13 | 11 | 12 | 8 | 10 | 11 | 8 | 12 | 13 | 12 | 13 | 12 | 13 |
| G5S0001 | 10 | 12 | 7 | 7 | 10 | 7 | 7 | 7 | 9 | 10 | 7 | 10 | 7 | 7 | 7 | 10 | 7 | 10 |
| D5S818 | 12 | 12 | 10 | 10 | 12 | 10 | 10 | 11 | 9 | 11 | 10 | 11 | 10 | 11 | 11 | 14 | 11 | 14 |

Note: The analysis was based on the 16 short tandem repeat loci and amelogenin with a human identification kit. Values in the chart represent the numbers of detected short tandem repeats at each locus.

**Supplemental Table 3. Genetic characteristics of published cases**

| Patient Number | Type of  Alport syndrome | Pathogenic gene | DNA variant | amino acid changes |
| --- | --- | --- | --- | --- |
| 1 | X-linked | COL4A5 | c.4271G>A | p.G1424E |
| 2 | X-linked | N/A | - | - |
| 3 | X-linked | N/A | - | - |
| 4 | N/A | - | - | - |
| 5 | X-linked | N/A | - | - |
| 6 | X-linked | COL4A5 | C.3120G>T | p.(G973V) |
| 7 | X-linked | COL4A5 | C.3710G>T | p.(G1170S) |
| 8 | N/A | - | - | - |
| 9 | autosomal recessive | COL4A4 | c.[3307G > A];[3307G > A] | p.G1102R |
| 10 | autosomal dominant | COL4A3 | C.2135g>T | p.G712V |
| 11 | autosomal dominant | COL4A3 | C.2135g>T | p.G712V |
| 12 | autosomal dominant | COL4A3 | C.2135g>T | p.G712V |
| 13 | X-linked | COL4A5 | - | - |
| 14 | X-linked | COL4A5 &COL4A4 | - | - |
| 15 | X-linked | COL4A5 |  |  |
| 16 | X-linked | COL4A5 | - | - |
| 17 | N/A | - | - | - |
| 18 | X-linked | COL4A5 | - | - |
| 19 | autosomal recessive | COL4A4 | c.[4708G>A];[3861delinsCTC] | p.(E1570K);p.(R1288Sfs*101) |
| 20 | N/A | - | - | - |
